# Evi1 forms a bridge between the epigenetic machinery and signaling pathways

**DOI:** 10.18632/oncotarget.304

**Published:** 2011-07-26

**Authors:** Akihide Yoshimi, Mineo Kurokawa

**Affiliations:** ^1^Department of Hematology & Oncology, Graduate School of Medicine, University of Tokyo, Tokyo, Japan

**Keywords:** Evi1, Leukemia, Polycomb, PTEN, Rapamycin

## Abstract

Recent studies have demonstrated the significance of the leukemia oncogene Evi1 as the regulator of hematopoietic stem cells and marker of poor clinical outcomes in myeloid malignancies. Evi1-mediated leukemogenic activities include a wide array of functions such as the induction of epigenetic modifications, transcriptional control, and regulation of signaling pathways. We have recently succeeded in comprehensively elucidating the oncogenic function of Evi1 in a model of the polycomb-Evi1-PTEN/AKT/mTOR axis. These results may provide us with novel therapeutic approaches to conquer the poor prognosis associated with Evi1-activated leukemia or other solid tumors with high Evi1 expression. Here, we review the current understanding of the role of Evi1 in controlling the development of leukemia and highlight potential modalities for targeting factors involved in Evi1-regulated signaling.

## INTRODUCTION

Ecotropic viral integration site 1 (Evi1) is a nuclear zinc finger protein that is essential for the proliferation and maintenance of hematopoietic stem cells (HSC) [1]. Clinically, activated Evi1 expression is observed in approximately 10% of patients with acute myeloid leukemia (AML) and is an independent factor associated with poor prognosis in AML [2-4]. Evi1 is located on chromosome 3q26, and the up-regulation of Evi1 expression was originally found to be a consequence of inv(3)(q21q26.2) or t(3;3)(q21;q26.2) [5,6]. In addition, we have recently shown that Evi1 is transcriptionally up-regulated by oncogenic MLL fusion proteins [7]; around half of the patients with AML having 11q23 rearrangements display high Evi1 expression [2]. Chromosomal abnormalities involving chromosome 7 are another candidate cause of Evi1 activation [2,8]. Molecularly, Evi1 has a variety of functions as an oncoprotein. Firstly, it regulates some signaling pathways. The most characterized function is the negative control exerted by Evi1 on TGF-β signaling through the repression of Smad3 function by physical interaction and recruitment of the corepressor CtBP [9,10]; this may contribute to the leukemogenic activity of Evi1 by promoting cellular proliferation and affecting cellular differentiation. Moreover, Evi1 exerts anti-apoptotic effects by suppressing the JNK1-mediated phosphorylation of c-Jun [11] or inhibiting interferon-α signaling through the regulation of the *PML* gene [12]. Secondly, Evi1 has been shown to act as a transcriptional factor and regulate the expression of several target genes by directly binding DNA through its proximal zinc finger domain and recognizing a consensus sequence consisting of GA(C/T)AAGA(T/C)AAGATAA-like or GACAAGATA-like motifs [13,14]. We and other groups have reported that Evi1 regulates GATA2 [14], PBX1 [15], and PML [12] transcription. Thirdly, recent studies have indicated that Evi1 actively induced epigenetic changes by interacting with various molecules. In this perspective, we will discuss the novel oncogenic functions of Evi1, particularly focusing on the relationship between leukemogenesis and epigenetics or signaling pathways.

## EPIGENETICS IN LEUKEMOGENESIS

Although cancer development has been considered a consequence of genetic changes, it has become increasingly evident that it also involves epigenetic changes that are mechanisms affecting gene expression profiles without alterations in the DNA sequence. Such mechanisms include DNA methylation, histone modification, and microRNA involvement. In hematological malignancies, recent findings of frequent mutations in epigenetic regulators such as EZH2 [16-18], DNMT3A [19-21], TET2 [22,23], ASXL1 [24], and UTX [25] support the idea that dysregulation of epigenetics plays a role in leukemogenesis [26,27]. For example, somatic mutations in EZH2 are frequently identified in myeloid malignancies, particularly in myelodysplastic syndrome and myeloproliferative neoplasms [16-18]. EZH2 is a core component of the polycomb repressive complex 2 (PRC2) and catalyzes histone 3 lysine 27 (H3K27) trimethylation in association with SUZ12 and EED. Another complex called PRC1 possesses histone 2A lysine 119 E3 ubiquitin ligase activities, and these histone modifications are essential for the silencing of polycomb target genes, which in turn regulate a broad array of biological processes such as the cell cycle, apoptosis, stem cell regulation, senescence, and cancer development [28-32]. Most of the missense mutations of EZH2 occurred in the CXC-SET domain and domain II, both of which are essential for the histone methyltransferase activity. Therefore, truncated or missense mutations in EZH2 observed in myeloid malignancies are supposed to be inactivating mutations [16], and EZH2 may be a tumor suppressor in this context. On the other hand, intact polycomb group (PcG) proteins function as oncogenic silencers in other contexts, as shown below.

## EVI1 LINKS THE EPIGENETIC MACHINERY TO THE REGULATION OF ITS TARGET GENE/SIGNALING PATHWAY

**Figure 1:**
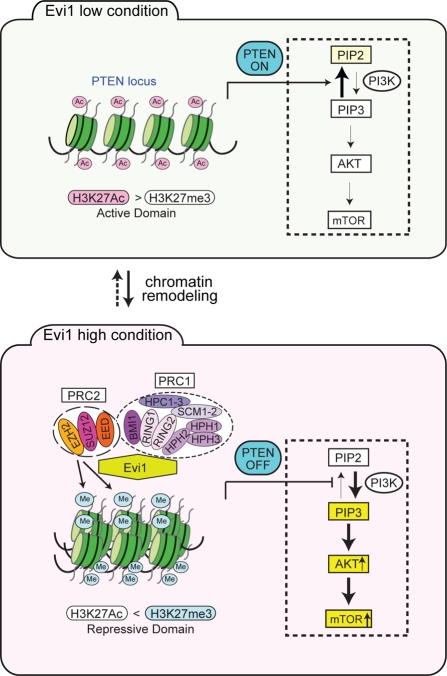
Evi1 induces epigenetic regulation on PI3K/PTEN/AKT/mTOR signaling In cells with high Evi1 expression, Evi1 recruits polycomb repressive complexes (PRC1 and PRC2) to the PTEN locus and epigenetically represses PTEN transcription, which in turn stimulates AKTand mTOR. These regulatory mechanisms are activated only in cells with high Evi1 expression.

We have recently found that Evi1 physically interacts with PcG proteins (EZH2, SUZ12, EED, BMI1, RING1, RING2, and HPH2) [33]. However, it appeared that Evi1 expression did not affect the global methylation status of H3K27 (data not shown). This finding prompted us to investigate specific target genes of Evi1 whose expressions were repressed in concert with PcG proteins. Our microarray data using primary bone marrow (BM) progenitors with forced expression of Evi1 and some sets of published gene expression data from AML samples [4,34] were combined and bioinformatically analyzed, leading to the identification of the tumor suppressor PTEN as a novel repressive target of Evi1. Chromatin immunoprecipitation (ChIP) assays using *Evi1*-transduced BM cells indicated that Evi1 induced H3K27 trimethylation marks on the *PTEN* genomic locus by recruiting PcG proteins. As PTEN is a well-established tumor suppressor and acts as a negative regulator of the PI3K/AKT pathway, and we found that Evi1 activated the AKT/mTOR signaling pathway through transcriptional repression of *PTEN*. The activation of this signaling is essential for Evi1-mediated leukemogenesis, as discussed later. Interestingly, RNAi-mediated knockdown of EZH2 restored PTEN expression and down-regulated the AKT/mTOR activities in *Evi1*-transduced BM cells, suggesting that Evi1 functions as a platform to recruit PcG proteins to the *PTEN* locus (Figure 1). In accordance with the aforementioned concept, the effects of EZH2 knockdown on PTEN expression were not observed in BM cells with low Evi1 expression. In other words, EZH2 can act as an oncoprotein in the presence of high Evi1 expression. Another example of the oncogenic function of EZH2 has been investigated in acute promyelocytic leukemia (APL). APL is induced by a differentiation block and an overgrowth of promyelocytes attributed to chromosomal translocation, leading to the production of fusion proteins such as PML/RARα and PLZF/RARα. These chimeric proteins have been shown to recruit PcG complexes to the target gene promoters through the RARα moiety and block cell differentiation [35,36]. Therefore, such oncogenic functions of PcG proteins can be therapeutic targets (Figure 1). In addition, we confirmed that *Evi1*-transduced BM cells show specifically increased sensitivity to a PRC2 inhibitor [3-Deazaneplanocin A (DZNep)] (data not shown).

Increasing evidence indicating the involvement of Evi1 in the epigenetic regulation of gene transcription has been recently accumulated. Lugthart et al. have shown that AML samples with activated Evi1 presented with a deregulated hypermethylation signature, possibly through physical interactions between Evi1 and the DNA methyltransferases DNMT3A and DNMT3B [37]. Their genome-wide DNA methylation profiling revealed about 300 promoters (out of the 14000 tested) with abundant cytosine methylation, and ChIP assays for the purpose of validation identified some Evi1 target genes such as *FAM83b*, *CRHBP*, *VPREB3*, and *IL11RA*. Given that EZH2 has been reported to interact with DNMT3A and DNMT3B [38], the two reports on Evi1 and histone/DNA methyltransferases may support each other in demonstrating that Evi1 repressed target gene transcription through, at least in part, PcG-DNMT complex-mediated epigenetic modifications.

Besides PcG proteins and DNMTs, we and other groups have demonstrated that Evi1 recruits epigenetic regulators for controlling transcription. For example, SUV39H1 and G9a are H3K9 methyltransferases associated with gene silencing [39,40], and they also interact with Evi1 [41-43]. Reporter assays have indicated that SUV39H1 synergistically suppressed the transcriptional activity of Evi1, and knockdown of SUV39H1 or G9a specifically reduced the colony-forming activity of *Evi1*-transduced BM cells, suggesting that H3K9 methyltransferases are actively involved in Evi1-associated oncogenic functions, possibly through the epigenetic repression of putative Evi1 target genes. In addition, Evi1 physically associated with the methyl-CpG binding protein 3b [44] and histone deacetylases 1 [45], both of which are the core components of the NuRD complex that harbors histone deacetylase activity and induces transcriptional repression [46,47]. Evi1 associates with Brahma-related gene 1 (*BRG1*; also known as SMARCA4 and SNF2β), a member of the SWI/SNF chromatin-remodeling complex [48]. In reporter assays, the authors have shown that Evi1 activated the E2F1 promoter in association with BRG1, which suggested that Evi1 enhanced cell cycling and cellular proliferation through the up-regulation of E2F1 by interacting with the SWI/SNF complex. Taken together, these data suggest that Evi1 has the potential to induce transcriptional repression by recruiting higher order chromatin remodeling complexes.

Along with chromatin remodeling factors, microRNAs play a part in epigenetic regulation, and recent findings have clarified that Evi1 regulates some microRNAs. Dickstein et al. have demonstrated that Evi1 silenced miRNA-124 expression through the methylation of CpGs located around miRNA-124 [49]. In addition, they have shown that Evi1 induction in Lin^−^ murine BM cells increased the expression of BMI1 and cyclin D3, whereas forced expression of miRNA-124 in *Evi1*-transduced murine BM cells restored their expression. Furthermore, Gomez-Benito et al. have recently shown that Evi1 directly bound to the miR-1-2 promoter region and up-regulated its expression, which may have a role in the Evi1-mediated proliferation activity, as shown by transient transfection assays using AML cell lines [50]. Likewise, Evi1 has been suggested to increase K-ras oncoprotein expression through the direct repression of miR-143 in colon cancer [51]. The authors argued that the Evi1/miR-143/K-ras axis contributed to colonic carcinogenesis by enhancing proliferation capacity and motility.

These data strongly suggest that Evi1 regulates target gene transcription by mobilizing epigenetic mechanisms.

## PI3K/AKT/MTOR SIGNALING IN EVI1-MEDIATED LEUKEMOGENESIS

**Figure 2:**
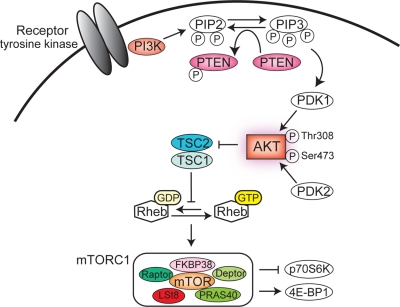
The PI3K/PTEN/AKT/mTORC1 pathway An activated tyrosine kinase receptor induces class IA PI3K activation, which in turn phosphorylates the lipid phosphatidyl-inositol bisphosphate (PIP2) to generate phosphatidyl-inositol triphoshate (PIP3). PIP3 recruits PDK1 and AKT to the plasma membrane, where PDK1 phosphorylates AKT on Thr308. The phosphorylation of AKT on Ser473 is mediated by PDK2 and controls AKT activity. AKT phosphorylates a wide variety of substrates, including FOXO transcription factors, Bad, p27kip1, p21Cip, GSK-3β, MDM2, and TSC2. AKT-driven TSC1/TSC2 complex inactivation leads to the accumulation of GTP-bound Rheb. Rheb-GTP then activates mTORC1. mTORC1 regulates protein synthesis by regulating p70S6K and 4E-BP1. In this pathway, PTEN phosphatase acts as a negative regulator through dephosphorylation of PIP3 at the 3’position.

Next, we focused on the PI3K/PTEN/AKT/mTOR signaling pathway in leukemia (overviewed in Figure 2; see other review articles such as [52-54] for more detailed information). As presented above, Evi1 recruited polycomb complexes to the *PTEN* genomic locus and activated the AKT/mTOR signaling pathway through the transcriptional repression of *PTEN* [33]. On the basis of these observations, we established a murine AML model with high Evi1 expression by BM transplantation and aimed to test the efficacy of the mTOR inhibitor rapamycin on the leukemia model in vivo. Administration of rapamycin significantly prolonged the survival of diseased mice, suggesting that the AKT/mTOR pathway played a role in the proliferation and survival of the Evi1-expressing AML cells. Moreover, we investigated the effects of rapamycin on other leukemia models established by transduction of the *AML1* mutant (AML1_S291fsX300) [55] and coexpression of TEL/PDGFβR and AML1/ETO [56] and found no changes in their survival; this demonstrated the specific sensitivity of Evi1-activated leukemia cells to rapamycin (Figure 1).

Extensive studies regarding PI3K/AKT/mTOR signaling and leukemia have demonstrated that PI3K/AKT signaling is frequently activated in AML [57,58]. In particular, AKT phosphorylation on Ser473 was detected in over half of the patients with AML [57]. As demonstrated by Tamburini et al. and Xu et al., downstream mTORC1 is constitutively activated in primary AML samples [58,59]. However, the mechanisms of PI3K/AKT/mTORC1 activation remain largely unclear. PTEN and SHIP are negative regulators of PIP3 and downstream AKT/mTOR signaling. Xu et al. have shown that PTEN expression was down-regulated in a few patients with AML, but PTEN expression levels were not inversely correlated with AKT phosphorylation levels for an unknown reason [58]. Gene mutation analysis of *PTEN* and *SHIP* has revealed that mutations in these genes are uncommon [60,61] although a *SHIP-V684E* mutation was found in one out of 30 patients with AML. This mutation reduced catalytic function and enhanced AKT phosphorylation, which may contribute to leukemogenesis [61]. On the other hand, PTEN phosphorylation levels have been analyzed in AML. Phosphorylated PTEN was positively correlated with AKT phosphorylation (Figure 2) and was found in 74% of patients with AML [62]. The authors have demonstrated that phosphorylated PTEN is a predictor of clinical outcome in patients with AML.

With regard to PI3K/PTEN/AKT signaling as a prognostic factor, some controversial results have been reported by several groups. Min et al. have shown that phosphorylation of AKT on Ser473 and Thr308 confers poor prognosis in AML [63]. Kornblau et al. have shown similar results using a Ser473-specific antibody ([64] and personal communication with S. Kornblau). Meanwhile, Tamburini et al. have evaluated as many as 188 patients and demonstrated that constitutive activation of PI3K was associated with better prognosis [57]. Furthermore, Gallay et al. have shown that AKT phosphorylation on Thr308, but not on Ser473, predicted an adverse outcome in AML [65]. These complicated data may be partly explained by the heterogeneity of leukemic cells used in evaluating PI3K/AKT phosphorylation [57,63,64]. Intracellular FACS staining and antibodies of high quality have enabled us to measure the phosphorylation status of a small amount of cells such as an immature population with CD34^+^, CD38^−^, and CD123^+^ phenotypes, wherein leukemia stem cells (LSCs) are supposed to be enriched [65,66]. Given that the PTEN/AKT/mTOR pathway plays an essential role in leukemia-initiating cells [67,68], the evaluation of this pathway in a purified leukemia population may provide some important insights. In addition, the aforementioned discrepancy may be attributed to the existence of cross-activation with other pathways or feedback systems in leukemic cells, as shown by Kornblau et al. that ERK and PKCα were more likely to be activated in leukemic cells with AKT phosphorylation than statistically expected ([64]; Excellent reviews on the cross-talk among the RAF/MEK/ERK, PI3K/PTEN/AKT/mTOR, and JAK/STAT pathways are available: [69,70]).

Our experimental model using murine primary BM has allowed the demonstration of one of the possible mechanisms of AKT/mTOR activation through *PTEN* repression in AML. We confirmed an inverse correlation between Evi1 and PTEN mRNA expression levels in human AML and chronic myeloid leukemia (CML) samples but have not checked the status of AKT/mTOR signaling. Thus, these data suggest the need for investigating whether Evi1-mediated AKT/mTOR activation in mice is recapitulated in human leukemia. Large scale studies applying a proteomic approach to human leukemic samples will provide comprehensive insight into the regulation of signaling pathways.

## HSC, LSC, AND THE EVI1-PTEN AXIS

*Pten* depletion in murine adult hematopoietic cells has caused short-term HSC expansion and long-term depletion of the HSC pool [67,68]. When *Pten*-deficient HSCs were transplanted into recipient mice, they could not reconstitute multilineage hematopoiesis. Moreover, *Pten* depletion has been shown to induce myeloproliferative neoplasms and their progression to acute leukemia, which may partly be a reflection of genomic instability [71]. Yilmaz et al. have demonstrated that rapamycin restored normal HSC function and effectively depleted leukemia-initiating cells simultaneously [67], suggesting that mTOR plays a significant role downstream of the PI3K/PTEN/AKT axis and can be an ideal therapeutic target.

Given that Evi1 is essential for HSC proliferation and myeloid leukemia cells [1], the Evi1-PTEN axis may potentially contribute to HSC and LSC regulation. Peng et al. have shown that BCR/ABL repressed *PTEN* in CML, and this repression was important for leukemogenesis [72,73]. The authors isolated Lin^−^, ckit^+^, and Sca1^+^ cells from BCR/ABL-induced and both BCR/ABL and PTEN-induced CML mice and transplanted the same number of each leukemic cell into the secondary recipient mice. The survival of the mice receiving LSCs transduced with both BCR/ABL and PTEN was significantly longer than that of the control mice, indicating that PTEN negatively regulated LSC functions. These results are consistent with our expression profiling of human CML samples showing an inverse correlation between Evi1 and PTEN levels and a tendency of Evi1 to be activated as the disease progresses from the chronic phase to blastic crisis [33]. Therefore, the Evi1-PTEN axis may play a role in LSC functions and the progression of leukemia.

## RAPAMYCIN MAY ACT AS A MULTI-FUNCTION AGENT IN A RECIPIENT OF ALLOGENEIC HSCT

**Figure 3:**
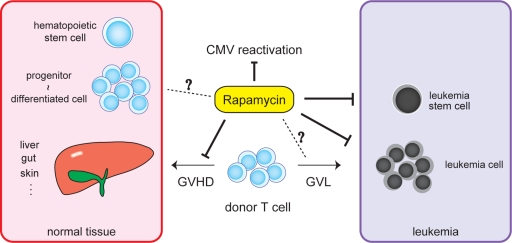
Rapamycin may act as a multi-function agent in a recipient of allogeneic hematopoietic stem cell transplantation Rapamycin exerts multiple functions including antineoplastic, immunosuppressive, and antiviral properties via inhibition of mTOR. In a recipient of allogeneic hematopoietic stem cell transplantation, rapamycin has a direct anti-leukemic potential. In addition, rapamycin can act as an immunosuppressive agent against graft versus host disease (GVHD) without impairing graft versus leukemia (GVL) effect. Rapamycin has been reported to reduce cytomegalovirus (CMV) reactivation after allogeneic stem cell transplantation. The effect of rapamycin on leukemia stem cells, GVL, or normal graft should be investigated more extensively.

Rapamycin binds to the FK506 binding protein 1A to form an immunosuppressive complex that inhibits mTOR and exerts antiviral, antifungal, antineoplastic, and immunosuppressive properties [74,75]. On the basis of the antineoplastic activities in preclinical studies, the efficacy of rapamycin (Sirolimus; Wyeth, Collegeville, PA, USA) and its analogs (rapalogs) has been investigated in clinical trials for the treatment of leukemia or lymphoma, and promising results have begun to be reported [76-86]. For instance, Recher et al. have shown that rapamycin achieved significant clinical response against four out of nine patients with either refractory/relapsed de novo AML or refractory secondary AML [87]. In addition to its antineoplastic efficacy, rapamycin suppresses T-cell proliferation/activation and has been used in the treatment of graft versus host disease (GVHD) after allogeneic hematopoietic stem cell transplantation (HSCT) [74,77,78,88-94]. In allogeneic HSCT for patients with lymphoma, a retrospective study comparing the outcome of GVHD prophylaxis between rapamycin-containing regimens and a combination of calcineurin inhibitor and methotrexate without rapamycin was conducted [95]. Intriguingly, the use of rapamycin significantly decreased disease progression without any differences in nonrelapse mortality in patients who underwent reduced-intensity conditioning regimens. This suggested that rapamycin can serve the dual purpose of an immunosuppressant and an antineoplastic agent. In addition, several reports have suggested the possibility that rapamycin exerts inhibitory effect on GVHD without interfering with graft versus leukemia (GVL) effect [96,97] (Figure 3). Moreover, Marty et al. reported that rapamycin-based GVHD prophylaxis reduced cytomegalovirus reactivation [98], which is still one of the challenging problems in HSCT because of the toxicity of cytomegalovirus itself or ganciclovir treatment-related toxicities such as myelosuppression and consequent risk of graft failure. On the other hand, the use of rapamycin or its analog, everolimus, may increase the risk of sinusoidal obstruction syndrome (veno-occlusive disease) under certain situations and lead to renal dysfunction, encephalopathy, and multiple organ failure, which is associated with high mortality [99,100]. Thus, the outcomes of large prospective trials that multilaterally evaluate the efficacy and toxicity of these agents in the treatment of leukemia and allogeneic HSCT are awaited.

## CONCLUSIONS AND FUTURE DIRECTIONS

Recent extensive studies have greatly increased our understanding of the roles of epigenetic regulation and signaling pathways in normal and malignant cells at the molecular level. These studies have demonstrated that the leukemia proto-oncoprotein Evi1 formed a bridge between the epigenetic machinery and signaling pathways and proposed therapeutic targets for the eradication of Evi1-related myeloid malignancies.

Recent studies have demonstrated that Evi1 is a strong inducer of epigenetic regulation of multiple genomic regions in hematopoietic cells. Clinical trials targeting patients with hematological malignancies have shown some efficacies and limitations of epigenetic agents such as histone deacetylase inhibitors and DNA methyltransferase inhibitors (reviewed in [101-105]). However, whether these drugs have the capacity to specifically counteract Evi1-mediated leukemogenic activity is unclear. Considering the marked clinical heterogeneity of hematological malignancies, accumulation of the results of epigenetic therapies on the basis of molecularly defined clusters will be essential for the appropriate use of epigenetic drugs. Efficient attenuation of the epigenetic regulators involved in Evi1-mediated transcriptional silencing potentially restores the expression of several possible tumor suppressors and improves the extremely poor prognosis of leukemia with activated Evi1.

Song et al. have recently reported that BMI1 induced epithelial–mesenchymal transition partially through transcriptional repression of *PTEN* in nasopharyngeal epithelial cells (NPECs) [106]. These results and those of our study have suggested that PcG proteins can target PTEN/AKT/mTOR signaling under specific circumstances. Our study proposes that one such condition in the hematopoietic system is high Evi1 activity because PcG proteins did not affect *PTEN* transcription in hematopoietic cells with low Evi1 expression. Meanwhile, it is yet to be determined whether anchor proteins like Evi1 are required in BMI1-mediated PTEN regulation in NPEC or other cells. Interestingly, a recent genome association study of nasopharyngeal carcinoma identified *MDS1/Evi1* on 3q26 as a susceptibility locus [107]. Given that Evi1 has been suggested to be implicated in several carcinomas other than hematopoietic malignancies [107-111], deep insight into the function of Evi1 with regard to epigenetic regulation and signaling pathways may contribute to the development of molecularly targeted therapies for solid tumors such as colon cancer, lung cancer, nasopharyngeal carcinoma, and ovarian cancer. Particularly, PI3K/AKT signaling is one of the most frequently and aberrantly regulated pathways in human cancer [54,112-114], and Evi1-mediated AKT activation has been demonstrated in colon cancer cells [109]. These reports have suggested that the Evi1-AKT axis is involved with several solid tumors. In addition, there is increasing evidence showing aberrantly activated PI3K/AKT/mTORC1 signaling in cancer stem cells, including leukemia as mentioned above [115-119]. Current clinical trials and studies using murine models have shown that rapamycin and rapalogs are less toxic to normal hematopoiesis than they are to malignancies and possibly less toxic to HSC than to LSC. More investigation for this scientific basis will enable us to develop rapamycin-containing therapy targeting AKT/mTORC1 activity in cancer stem cells.
